# Wireless Power Transfer to Millimeter-Sized Gastrointestinal Electronics Validated in a Swine Model

**DOI:** 10.1038/srep46745

**Published:** 2017-04-27

**Authors:** Abubakar Abid, Jonathan M. O’Brien, Taylor Bensel, Cody Cleveland, Lucas Booth, Brian R. Smith, Robert Langer, Giovanni Traverso

**Affiliations:** 1 Department of Electrical Engineering, Massachusetts Institute of Technology, Cambridge, MA 02139, USA; 2 The David H. Koch Institute for Integrative Cancer Research, Massachusetts Institute of Technology, Cambridge, MA 02139, USA; 3 Charles Stark Draper Laboratory, Cambridge, MA 02139, USA; 4 Department of Chemical Engineering, Massachusetts Institute of Technology, Cambridge, MA 02139, USA; 5 Division of Gastroenterology, Brigham and Women’s Hospital, Harvard Medical School, Boston, MA 02115, USA; 6 Institute for Medical Engineering and Science, Massachusetts Institute of Technology, Cambridge, MA 02139, USA

## Abstract

Electronic devices placed in the gastrointestinal (GI) tract for prolonged periods have the potential to transform clinical evaluation and treatment. One challenge to the deployment of such gastroresident electronics is the difficulty in powering millimeter-sized electronics devices without using batteries, which compromise biocompatibility and long-term residence. We examined the feasibility of leveraging mid-field wireless powering to transfer power from outside of the body to electronics at various locations along the GI tract. Using simulations and *ex vivo* measurements, we designed mid-field antennas capable of operating efficiently in tissue at 1.2 GHz. These antennas were then characterized *in vivo* in five anesthetized pigs, by placing one antenna outside the body, and the other antenna inside the body endoscopically, at the esophagus, stomach, and colon. Across the animals tested, mean transmission efficiencies of −41.2, −36.1, and −34.6 dB were achieved *in vivo* while coupling power from outside the body to the esophagus, stomach, and colon, respectively. This corresponds to power levels of 37.5 μW, 123 μW and 173 μW received by antennas in the respective locations, while keeping radiation exposure levels below safety thresholds. These power levels are sufficient to wirelessly power a range of medical devices from outside of the body.

Wireless medical electronics have become integral diagnostic and therapeutic tools for clinicians in a variety of applications, from pacemakers used by cardiologists to neural probes used in brain-machine interfaces^1^. However, to the extent that wireless electronic devices are used in gastrointestinal (GI) applications, they are generally not designed to reside for prolonged periods of time inside of the body. For example, wireless capsule endoscopy and electronic drug delivery devices are both designed to stay inside the body for less than 30 hours, consistent with the range of regular transit time for objects in the GI tract^2^.

The lack of GI-resident electronics remains despite the fact that GI-resident *platforms*, which are designed to stay inside the GI tract for weeks or longer, have been previously developed in the form of balloons^3^, stents^4^, and more recently systems for drug delivery^5^. One key challenge to the deployment of GI-resident electronics is powering them for the lifetime of the devices. Typical power sources, such as batteries, come with disadvantages such as added bulk and a compromise in biocompatibility with the potential for mucosal injury^6^,^7^. These obstacles have prompted researchers to consider electronics that are powered by glucose^8^ or acid^9^,^10^ in the gastric environment. However, such electrochemical batteries have limited lifetimes^9^,^10^ due to the finite size of the electrodes, making them less suited for powering GI-resident electronics for an extended period of time.

A more promising solution is to power devices from *outside* of the body, relying on wireless power transfer through the tissue to GI-resident electronics. Near-field coupling is a well-established technique used to deliver power wirelessly in medical applications, such as neural probes and cochlear implants^11^. However, because near-field electromagnetic fields decay as a function of distance squared, near-field coupling is only efficient in the regime where the antenna size is on the same order as separation between the antennas^12^. For some gastric electronic applications this may be the case^13^ – and near-field power can be used to deliver power efficiently – but for most ingestible devices the electronics are constrained to be 6–8 mm in diameter to fit inside an ingestible capsule while the separation through tissue is an order of magnitude larger: around 6 centimeters. The maximum theoretical efficiency of near-field coupling at this distance is far too low to power medical devices (see Supplementary Materials for detailed calculations).

Recently, mid-field coupling has been proposed as an alternative way to deliver power into deep micro-implants by focusing propagating fields^14^. By operating at higher frequencies (in the low GHz range, as compared to MHz for near-field coupling), mid-field coupling has been shown to deliver power at efficiencies 2–3 orders of magnitude higher than conventional near-field coupling in cardiac and neural applications^15^.

While there is prior work in the design of antennas for ingestible capsules at higher frequencies, where propagating electromagnetic fields play a significant role^16^,^17^, these antennas have been used for communication and have not been investigated for wireless power delivery. In this paper, we show that two loop antennas operating at 1.2 GHz, in the mid-field coupling regime, can be used to deliver power efficiently to ingestible GI-resident electronics placed inside a pig from outside of the pig’s body. We design transmit and receive antennas through full wave simulations and establish safety thresholds. Simulations are validated through *ex vivo* measurements before antennas are placed inside anesthetized pigs to measure *in vivo* power transfer efficiencies.

## Methods

### Simulation and *Ex Vivo* Characterization

**Figure 1:**
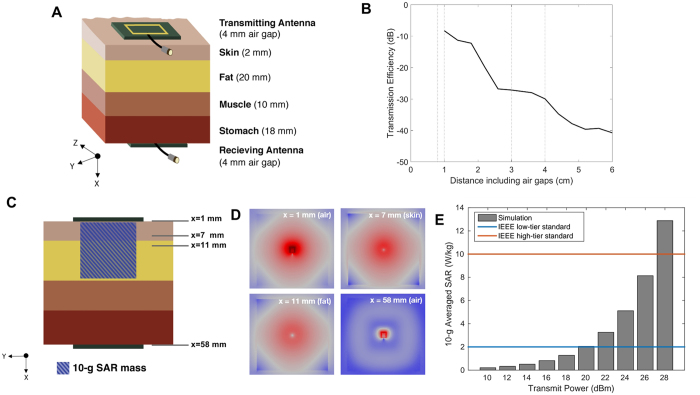
Simulations were performed to estimate mid-field coupling efficiency and specific absorption rate (SAR) of radiation. (**A**) The multilayer tissue model (not drawn to scale) used in simulation studies consists of 2 mm of skin, 20 mm of fat, 10 mm of muscle, and 18 mm of stomach tissue arranged as shown. Antennas are placed 4 mm away on either side of the tissue model. (**B**) The simulated transmission coefficient or S_21_ parameter is shown as a function of distance through the multilayer tissue model. The dotted lines show the transitions between different tissue. From left to right, they are: air-skin, skin-fat, fat-muscle, and muscle-stomach tissue. The efficiency across the complete multilayer tissue is about −41 dB. (**C**) For the SAR calculations, a 10-g mass of tissue (striped blue; a cube of tissue of side length 2.15 cm, not drawn to scale), directly under the center of transmitting antenna, was used. The location of the tissue was based on inspection of the SAR in different planes in the x-axis, as indicated by the labels on the right side of the tissue model. (**D**) The relative magnitude of SAR is shown in different slices (planes in the x-direction). Each slice is labeled with its distance from the transmitting antenna. Red indicates regions of high absorption, while blue indicates regions of low absorption. In the slices that are outside of the tissue (at x = 1 and x = 58 mm), the magnitude of the mean-squared electric field is plotted instead of the absorption. The color scale is for qualitative comparison within a slice only, as the scale differs among slices. (**E**) Averaged value of SAR is as a function of the power delivered into the transmitting antenna. IEEE sets a low-tier (blue) and high-tier (red) standard for safety limits.

Simulations were conducted in COMSOL (Burlington, MA) to estimate the efficiency of wireless power transfer between two small square loop antennas each 6.8 mm in length. The antenna length was chosen to fit on a printed circuit board inside the largest ingestible capsule (000 capsule), which is 9.5 mm in diameter. A 5.8-cm thick, multilayer tissue model consisting of air gaps, skin, fat, muscle, and stomach tissue was constructed (Fig. 1A). The dielectric properties of tissue were obtained from an existing database^18^.

Two antennas were placed on either side of the multilayer tissue model, and the power transfer efficiency was recorded as a function of distance at a frequency of 1.2 GHz. The distance was varied by moving the receiving antenna through the multilayer tissue model, while keeping a fixed, 4-mm air gap around it. The power efficiency was quantified by plotting the transmission coefficient, S_21_ parameter, which represents the fraction of power that is fed from a port into a transmitting antenna that is picked up by a receiving antenna and absorbed by its associated port.

Simulations were also performed to determine the maximum power that could be radiated into the stomach before specific absorption rate (SAR) limits in tissue were exceeded. In this work, SAR limits identified by IEEE^19^ were used. These limits apply to SAR averaged over a 10-g tissue sample (a cubical sample 2.15 cm on each side) where the electric field is the strongest. In our case, as shown in Fig. 1C, the sample was centered directly under the antenna as it was assumed (and later verified) that the electric fields would be highest in that volume. The absorbed radiation for a variety of external power levels was simulated, and the maximal amount of power that could be coupled safely into the tissue was identified.

**Figure 2:**
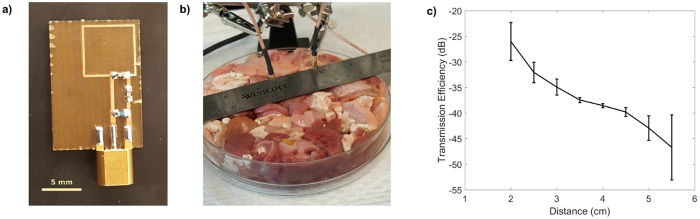
Tests in stomach tissue identified optimal antennas for *in vivo* work. (**a**) 1.2 GHz small loop antenna fabricated on FR4 substrate with lumped components soldered onto the board. (**b**) Antennas were encapsulated and then placed in ground porcine stomach tissue, separated at various distances to characterize the transmission efficiency. (**c**) The measured transmission coefficient or S_21_ parameter is shown as a function of distance. Six measurements were taken for each distance in different parts of the tissue. The error bars shown the maximum and minimum values recorded from the six measurements at each distance. The solid line passes through the mean of the maximum and minimum measurement.

After verifying through simulation that antennas operating at 1.2 GHz could transfer power at a reasonable efficiency through the stomach, a series of small loop antennas were fabricated and impedance matched to resonate at 1.2 GHz (see Fig. 2a). Resonance and impedance matching was achieved by placing 2.2 pF surface-mount capacitor in parallel with the loop antenna, followed by an L-network consisting of a series 33 pF capacitor and a shunt 1 nH inductor. Small MMCX connectors with footprints of 7.4 by 4.5 mm were attached to the boards.

The antennas were coated in a layer of 5-minute epoxy^20^ (Devcon) and then encapsulated in a layer of PDMS (Sylgard^®^ 184 Silicone Elastomer) to ensure stability in tissue and the GI environment. The PDMS coating was created by suspending the circuit board into a 3D-printed plastic mold with a 9-mm inner diameter. The PDMS was cured at room temperature for 48 hours. This created a coating of PDMS between 1–4 mm thick around the circuit board.

The encapsulated antennas were placed in porcine stomach tissue ground into roughly 1 cm^3^ chunks (see Fig. 2b) to characterize *ex vivo* wireless power transfer. An HP 8753 C vector network analyzer (VNA) was used to record the transmission coefficient between the antennas, which were held apart at different distances within the tissue. Six measurements were taken at each distance; between each measurement, the antennas were removed from the tissue and placed in a different part of the tissue to account for the heterogeneity in stomach tissue. Antennas validated *ex vivo* were then used for the *in vivo* study.

### *In Vivo* Measurements

All animal work was approved by the Committee on Animal Care at the Massachusetts Institute of Technology, and all experiments were carried out in accordance with the guidelines and regulations of approved protocol 1013–094–16.

Five healthy female Yorkshire pigs weighing 60–75 kg were used for this study. Animals were fasted overnight prior to the procedure. On the day of the procedure the morning feed was held. The animals were sedated through intramuscular dosing of Telazol (tiletamine/zolazepam) 5 mg/kg, xylazine 2 mg/kg, and atropine 0.04 mg/kg. Esophageal intubation and placement of an esophageal overtube (US Endoscopy) was performed.

One antenna was attached to an endoscope (Pentax) and placed in the esophagus. Once the antenna had been situated in a stable position inside of the esophagus, 30 centimeters from the mouth, it was held still while the other antenna was held outside of the body, pressed against the skin, and adjusted to determine the position and orientation for optimal power transfer efficiency. The device inside the body was not intentionally moved or rotated to mimic actual deployed devices, which could not be adjusted once inside the body. The same procedure was repeated with the antenna placed inside the stomach (75 centimeters from the mouth) and inside the colon. In the latter case, the endoscope was placed through the rectum, 25 cm from the anus.

**Figure 3:**
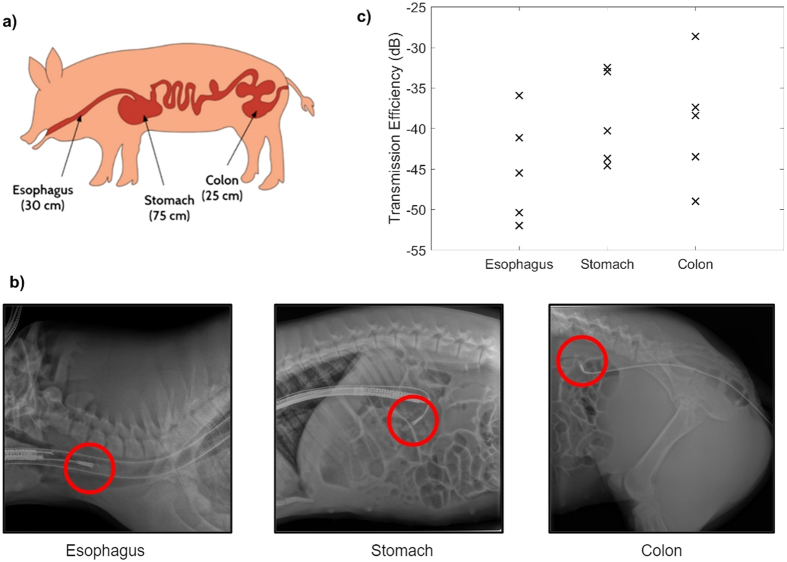
Receiving antennas were placed endoscopically in three locations in anesthetized swine for *in vivo* measurements. The locations are shown schematically in (**a**) and under x-rays (**b**). The locations were 30 cm through the mouth into the esophagus, 75 cm through the mouth into the stomach, and 25 cm through the rectum into the colon. (**c**) The measured transmission efficiency or S_21_ parameter is shown in the esophagus, stomach, and colon in each of the five animals. Within each animal, the standard deviation in measured efficiency was minimal (less than 0.2 dB) during the optimal 6-second observation window and is not shown in the plot.

The power transfer efficiency was measured by observing the transmission coefficient at the resonant frequency. Once the external antenna had been placed next to the skin, a continuous 60-second measurement of the S_21_ parameter was made at 4 Hz. Within this measurement, the 6-second window with the highest average reading was identified and the average and standard deviation was recorded. Representative x-rays and optical images were taken to indicate the position of the antenna inside of the animal, as shown in Fig. 3a,b. This was repeated for the stomach and colon.

## Results

### Simulations and *Ex Vivo* Characterization

The mid-field coupling efficiency as a function of distance is shown in Fig. 1B for the multilayer tissue model. The plot shows a steady and expected decrease in simulated S_21_ magnitude as a function of distance. At 5–6 cm, the attenuation between the antennas is around −40 dB. Simulations suggest that most of the attenuation is likely dielectric losses in tissue; the path loss is less than −10 dB.

IEEE sets two thresholds for the maximum radiation that can safely be absorbed by tissue: a high-tier threshold for radiation emitted by a medical device that is well-characterized and a low-tier threshold for experimental, laboratory devices. For gastric electronic devices that are to be used in clinical settings, the high-tier threshold is more appropriate. Based on this threshold, simulations showed that the power level for the transmitting antenna should not exceed 27 dBm (500 mW), as plotted in Fig. 1E.

The full-wave simulation also calculated the specific absorption rate at points inside the multilayer tissue model. These are shown qualitatively in Fig. 1D. The simulations show that the highest absorption rates are directly beneath the antennas, justifying our choice of the mass over which to average SAR.

*Ex vivo* power transfer efficiency of the antennas is shown in Fig. 2c. The maximum and minimum S_21_ value from the 6 measurements are indicated by the error bars. At a distance of 5 cm, the loss between the antennas never exceeded −47 dB, although at 5.5 cm, losses of up to −54 dB were measured. These losses are about ten times higher than those predicted in simulations. This is likely due to a combination of factors, including losses in the antenna dielectric, as well as the different composition of ground stomach tissue as compared to the multilayer tissue model.

### *In Vivo* Measurements

The average efficiency of power transfer in each location across the five animals is shown in Fig. 3c. The variation within each animal is not shown because the standard deviation in measured efficiency was minimal (less than 0.2 dB) during the optimal 6-second observation window. Outside of the window, there was some variation, but this was attributed to disturbances in the position of the endoscope.

For devices in the stomach, the values of the transmission loss varied from −32.5 dB to −44.6 dB, with a mean value of −36.1 dB, similar to simulated results. As compared to recordings in the stomach, recordings in the esophagus showed lower mean transmission efficiency (−41.2 dB), while those in the colon showed about the same mean transmission efficiency (−34.6 dB) as in the stomach, but increased variability.

There is significant variability in measured transmission efficiency across animals. This is due likely in part to anatomical variations among the swine and in part to variations in antenna position inside the animal body, as it was difficult to control the depth, lateral position, and orientation of the antenna after it had reached a stable position within the esophagus, stomach, or colon. Despite these variations, measured efficiencies are consistently 1–2 orders of magnitude higher than the theoretical near-field efficiency for similarly-sized antennas (see Supplementary Materials).

Taking into account the simulated safety limits, these results suggest that one is able to deliver on average approximately −9.1 dBm (123 μW) of power to antennas inside the stomach. This value is calculated by starting with SAR simulations, which limit the external antenna to transmit up to 27 dBm of power. The attenuation through the stomach tissue, as measured by the transfer coefficient, has a mean value of −36.1 dB, which gives a net result of −9.1 dBm as the amount of power received.

The mean and standard deviation of the power levels that can be delivered to antennas at each location in the GI tract are recorded in Table 1, and the power consumption of representative medical devices is included for comparison.

## Discussion

Our work demonstrates that mid-field powering is a viable method to deliver wireless power efficiently from outside of the body to electronics that reside for long periods of time in the GI tract. Despite some variability in measurements, the efficiencies that we recorded are consistently higher than theoretical maximum efficiencies using conventional near-field power transfer with antennas of the same size, while remaining under safety thresholds.

In further work, we would like to expand on these measurements by characterizing the effects of animal size, antenna depth, lateral position, and orientation on transmission efficiency. In addition, although operating in mid-field frequencies, we have not attempted to focus propagating fields, as has been done in some previous studies^14^,^15^. Focusing the propagating fields may lead to further increases in efficiency.

Wireless powering from outside of the body can thus enable low-power, millimeter-sized gastric electronics, such as imagers, gastric pacemakers, and a variety of radio-enabled sensors. Devices with higher power budgets, such as locomotive implants and electrical stimulators, may also be within reach if the propagating fields are focused or multiple or larger antennas are used, enabling clinicians to have access to a variety of diagnostic and therapeutic tools for the gastrointestinal system.

## Additional Information

**How to cite this article:** Abid, A. *et al*. Wireless Power Transfer to Millimeter-Sized Gastrointestinal Electronics Validated in a Swine Model. *Sci. Rep.* **7**, 46745; doi: 10.1038/srep46745 (2017).

**Publisher's note:** Springer Nature remains neutral with regard to jurisdictional claims in published maps and institutional affiliations.

## Supplementary Material

Supplementary Information

## Figures and Tables

**Table 1:** Power characteristics of GI-resident electronics.

GI Tract Location	Mean Power delivered^a^
Esophagus	37.5 μW
Stomach	123 μW
Colon	175 μW
Electronic Device	Power consumption
CMOS Imager^21^	3.4 μJ per frame
Low-power pacemaker^22^	8 μW
Bluetooth-enabled temperature sensor^23^	30 μW transmitting at 0.1 Hz
Locomotive implants^24^	250 μW at 0.53 cm/s

^a^Means were calculated after converting from logarithmic units to linear units. More information, including standard deviations, are included in the Supplementary Materials.
