# Supplementary material for: Wireless Power Transfer to Millimeter-Sized Gastrointestinal Electronics Validated in a Swine Model
Source: Sci Rep. 2017 Apr 27;7:46745. doi: 10.1038/srep46745 (PMC5406829; doi:10.1038/srep46745)
Supplement: Supplementary Information [file srep46745-s1.pdf]

Wireless Power Transfer to Millimeter-Sized Gastrointestinal Electronics Validated in a Swine Model

Abubakar Abid[a,b,c], Jonathan M. O'Brien[c], Taylor Bensel[b,d], Cody Cleveland[b,d,e], Lucas Booth[b,d], Brian R. Smith[c], Robert Langer[b,d,f], Giovanni Traverso[d,e]

[a]Department of Electrical Engineering, Massachusetts Institute of Technology, Cambridge, MA 02139

[b]The David H. Koch Institute for Integrative Cancer Research, Massachusetts Institute of Technology, Cambridge, MA 02139

[c]Charles Stark Draper Laboratory, Cambridge, MA 02139

[d]Department of Chemical Engineering, Massachusetts Institute of Technology, Cambridge, MA 02139

[e]Division of Gastroenterology, Brigham and Women's Hospital, Harvard Medical School, Boston, MA 02115

[f]Institute for Medical Engineering and Science, Massachusetts Institute of Technology, Cambridge, MA 02139

Correspondence:

[*]Correspondence should be addressed to Giovanni Traverso and Robert Langer

Dr. Giovanni Traverso
Division of Gastroenterology
Brigham and Women's Hospital
75 Francis St, Thorn 14,
Boston, MA 02115
Tel: 617-417-8061
Fax: 617-500-0631
Email: gi_lab@mailworks.org

Professor Robert Langer
Koch Institute for Integrative Cancer Research, Room 76-661
Massachusetts Institute of Technology
77 Massachusetts Avenue
Cambridge, MA 02139
Tel: 617-253-3107
Fax: 617-258-8827
Email: rlanger@mit.edu

SUPPLEMENTARY MATERIALS

Theoretical Limits of Resonant Inductive (Near-Field) Coupling

While near-field coupling has been demonstrated in many medical applications, it is only effective when the distance between the transmitting and receiving coils is on the order of the distance between the coils. For separation distances that are much larger than the coil size (as will be the case for ingestible, GI-resident devices powered from outside the body), near-field wireless power transfer is considered inefficient.

Here, this is demonstrated by establishing the theoretical near-field efficiency between two coils that are 6.8 mm in diameter and 6 cm apart. A circuit representation of resonant inductive power is shown below (Supplementary Fig. S1).

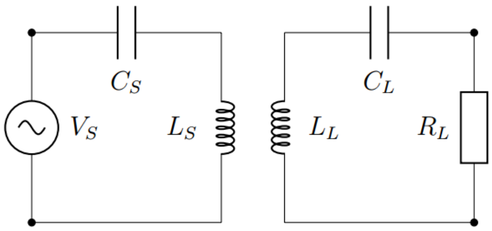

Figure S1

The efficiency of this circuit is given by the expression below (see [1] for derivation):

$$\eta = \frac{(kQ)^2}{(kQ)^2 + 2(1 + \sqrt{1 + (kQ)^2})}$$

where *Q* is the quality factor of each coil and *k* is the coupling factor between the two coils. This expression makes use of the assumptions that (1) the coils resonate at the same frequency, (2) the coupling factor is small ($k^2 << 1$), and (3) the load resistance has been optimized for maximum efficiency. In reality, the efficiency will be lower, but this expression provides an upper bound.

The quality factor for an inductor with inductance *L* and series resistance *R* at its resonant frequency, ω_0, is defined [1] as:

$$Q = \frac{L\omega_0}{R}$$

The coupling coefficient between the two coils depends on the radius of the coils and the distance between them. For two single-turn coils, it can be approximated [2] as:

$$k = \frac{1}{[1 + (\sqrt[3]{2}d/r)^2]^{3/2}}$$

Substituting the size of and distance between the coils, the efficiency curve can be plotted as a function of distance and quality factor, as in the figure below (Supplementary Fig. S2). The quality factor is assumed optimistically to be as high as 70 [3].

It is apparent from the figure that for even very high-quality coils, the theoretical efficiency at near-field at a distance of 6 cm is -50 dB or less, lower than the simulated (and *in vivo* measured) efficiencies in the mid-field regime. For more typical values of the quality factor, the near-field efficiencies are even lower. This is the motivation to use mid-field coupling as opposed to conventional near-field coupling.

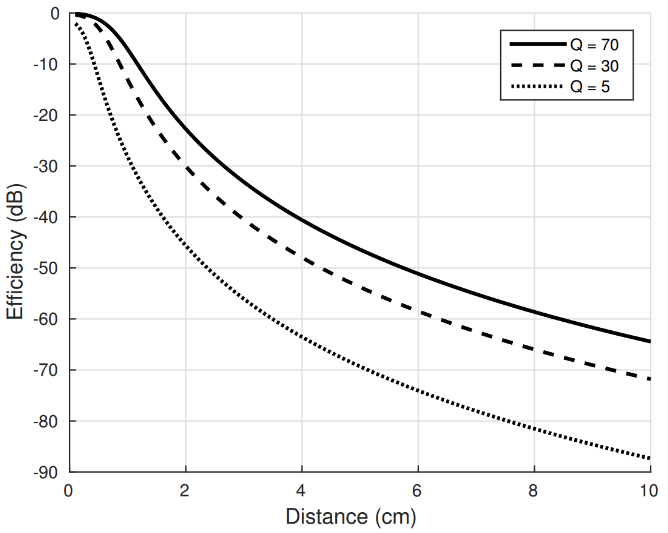

Figure S2

Reflection (S_{11}) Characteristics of Antennas

The mid-field antennas were fabricated and matched to resonate at about 1.2 GHz. The reflection coefficient or S_{11} parameter identifies the frequency at which an antenna resonates, and is shown here (Supplementary Fig. S3) for one of the antennas that were fabricated. The S_{11} parameter was measured by a VNA when the encapsulated antenna was in free space, as well as when the encapsulated antenna was placed in chopped porcine stomach tissue.

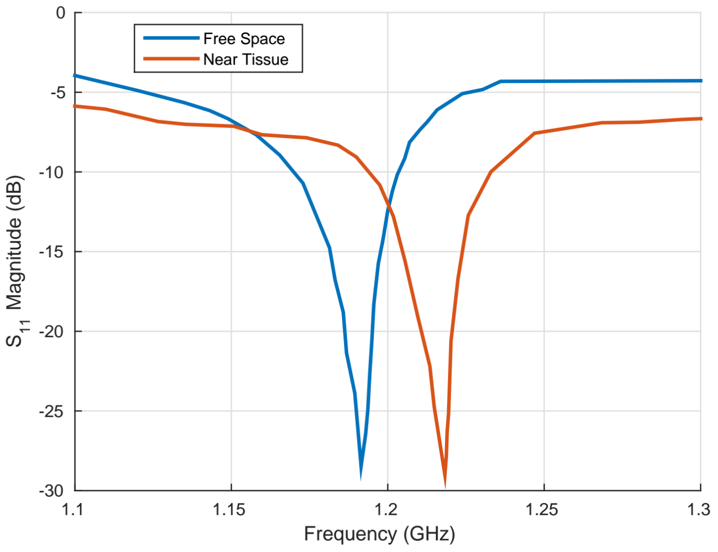

Figure S3

While there is a small shift in the resonant frequency when the antenna is placed in tissue, the reflection coefficient remains about -30 dB (0.1%). Thus, about 0.1% of the incoming power is reflected back to the VNA – the rest is either radiated away or dissipated in the antenna, cables, and connectors.

Mean and Standard Deviations of In Vivo *Study*

The mean power levels were calculated by first converting the measured transfer coefficients across the five animals from logarithmic ratios to linear ratios. The linear ratios were all multiplied by 500 μW (equal to 27 dBm, the transmit power level set by the SAR limit) to obtain the power levels that could be received by antennas placed in each of the five animals. For each location, the mean of the five measurements was calculated and is shown in Table 1.

Along with the mean power levels, the standard deviations can also be calculated. The standard deviations in the esophagus, stomach, and colon are 52.1, 130, and 286 μW. The magnitudes of the standard deviations are quite large because the transfer coefficient measurements across the five animals are spread out over an order of magnitude. As discussed in the paper, this is most likely due to anatomical variations among the swine and to variations in the position and orientation of the antenna inside the animal body.

References for Supplementary Material

[1] Rikard Vinge. “Wireless energy transfer by resonant inductive coupling.” Master’s thesis, Chalmers University of Technology, 2015.

[2] Jose Oscar Mur-Miranda, Giulia Fanti, Yifei Feng, Keerthik Omanakuttan, Roydan Ongie, Albert Setjoadi, and Natalie Sharpe. Wireless power transfer using weakly coupled magnetostatic resonators. In *2010 IEEE Energy Conversion Congress and Exposition*. Institute of Electrical & Electronics Engineers (IEEE), sep 2010

[3] A. Eroglu. Planar inductor design for high power applications. *Progress In Electromagnetics Research B*, 2011
